# Finding and Testing Network Communities by Lumped Markov Chains

**DOI:** 10.1371/journal.pone.0027028

**Published:** 2011-11-03

**Authors:** Carlo Piccardi

**Affiliations:** Department of Electronics and Information, Politecnico di Milano, Milano, Italy; University of Zaragoza, Spain

## Abstract

Identifying communities (or clusters), namely groups of nodes with comparatively strong internal connectivity, is a fundamental task for deeply understanding the structure and function of a network. Yet, there is a lack of formal criteria for defining communities and for testing their significance. We propose a sharp definition that is based on a quality threshold. By means of a lumped Markov chain model of a random walker, a quality measure called “persistence probability” is associated to a cluster, which is then defined as an “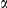-community” if such a probability is not smaller than 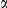. Consistently, a partition composed of 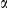-communities is an “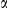-partition.” These definitions turn out to be very effective for finding and testing communities. If a set of candidate partitions is available, setting the desired 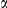-level allows one to immediately select the 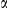-partition with the finest decomposition. Simultaneously, the persistence probabilities quantify the quality of each single community. Given its ability in individually assessing each single cluster, this approach can also disclose single well-defined communities even in networks that overall do not possess a definite clusterized structure.

## Introduction

Complex networks are currently one of the most extensively studied subjects in the field of applied mathematics. In the last fifteen years, a huge number of theoretical results have been put forward, and almost any field of science and technology has benefit from the application of such results to specific problems [1]–[4].

One of the most promising but challenging tasks in network science is *community analysis*, which is aimed at revealing possible partitions of a network into subsets of nodes (*communities*, or *clusters*) with dense intra- but sparse inter-group connections. Finding and analyzing such partitions often provides invaluable help in deeply understanding the structure and function of a network, as widely demonstrated by several case studies in social sciences [5], [6], biology [7], ecology [8], economics [9], or information science [10], [11], just to name a few.

Despite the abundance of contributions on this subject (see [12] for a thorough survey), the issue of community analysis cannot be considered satisfactorily solved. First of all, finding communities is a computationally hard task, because the “best” partition must be sought for in a set whose cardinality grows faster than exponentially with the number of nodes. The exhaustive enumeration of the partitions is thus impossible, and heuristic techniques must be employed. Secondly, and perhaps more important, there is no widespread consensus on formal criteria for defining communities and for testing their significance [12]. When a subnetwork can actually be considered to form a community, namely a group of nodes with comparatively strong internal connectivity? Many contributions, mostly coming from social sciences, computer sciences, and physics, have tried to answer this question in various ways, over the years (e.g., [13]–[16]). Probably the most important attempt was put forward by Newman and coworkers [5], [17], [18], who defined a quality index called *modularity* which quantifies, for a given partition of the network into candidate communities, to what extent the distribution of the intra-/inter-community edges is anomalous with respect to a suitably defined random network. Since high modularity values are obtained in presence of groups of nodes with comparatively large intra-community edge density, maximizing modularity should put in evidence the “best” partition. This method has been proven successful in many circumstances but, on the other hand, it has been widely demonstrated that, due to intrinsic limitations, it does not necessarily always yield a significant partition [12], [15], [19]. And even when it does, it quantifies the quality of a partition but not of each individual community. For that, many other methods for community analysis have been put forward in the last few years, trying to simultaneously finding a meaningful network partition and assessing its significance (we recall, e.g., [20]–[22]).

This paper introduces a sharp definition of community which is based on a quality threshold. More precisely, once a level 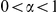 is specified, a node cluster is defined to be an 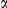 *-community* if the probability that a random walker, which is currently in one of the cluster's nodes, remains in the cluster in the next step is not smaller than 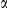. Such a probability is obtained from an approximate *lumped Markov chain* model of the random walker (i.e., a reduced-order Markov chain in which the communities of the original network become nodes) which is easily derived from the original (high-order) Markov chain model. Consistently, a partition composed of 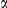-communities is defined to be an 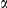 *-partition*.

If equipped with an effective method for generating a set of “good” candidate partitions, the notions of 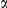-community and 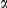-partition provide a framework for simultaneously finding communities and testing their significance. For that, the desired quality level 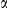 is first fixed. Then, a family of partitions is derived and each partition is immediately checked to assess whether it is formed by 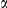-communities. This allows one to identify the 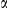-partitions, and to select one of them. Typically, one searches for communities which are at the same time small (to effectively decompose the network) and significant (with much more internal than external connectivity). For that, a guideline is that of selecting, among the available 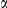-partitions, the one with the largest number of communities.

But the notion of 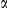-community can also be useful in a partially different way. It may happen that, for a given quality level 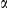, no 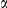-partitions are found. Yet, one or a few 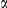-communities could exist. They correspond to strongly connected groups of nodes, even in a network which, overall, does not possess a definite clusterized structure. Or, finally, one can assess the significance of the results of a single-partition method, such as modularity optimization [5], and obtain an immediate assessment of the 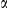-quality of each single community and, consequently, of the entire partition.

In the paper, we first introduce the lumped Markov chain model of the random walker and define the notions of persistence probability, 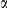-community, and 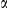-partition. Testing the 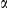-quality of a given community or partition turns out to be extremely parsimonious in computational terms. Then we analyze the problem of finding communities in a given network. For that, we propose an effective algorithm for deriving a meaningful set of partitions, among which the “best” one will be selected. The algorithm, which applies hierarchical cluster analysis, is again based on the Markov chain model of a random walker and, consequently, it involves a notion of similarity/distance among nodes which is consistent with the quality criterion above introduced. The results of the application of the above approach to both synthetic and real-world networks are discussed. We finally compare this approach, which can be applied to fully general networks (i.e., directed and weighted), with other community analysis methods having a similar philosophy.

## Methods

### Networks, 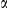-Communities, and 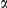-Partitions

Consider a network with nodes 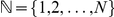 and 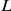 edges. In the most general case the network is directed and weighted, and we denote by 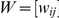 the 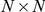 weight matrix, where 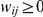 is the weight of the edge 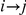. The connectivity matrix 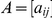 is the 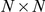 binary matrix where 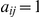 if 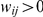, and 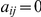 otherwise. If the network is actually undirected we have 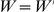 and 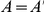, and if it is unweighted we let 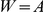 (i.e., all weights equal to 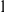). Since connectedness is typically required for communities ([12], p. 84), we naturally assume that the network is strongly connected (e.g., [3]), namely there exists an oriented path from any 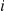 to any 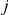. If this is not the case, namely the network is disconnected, each strongly connected component must be separately analyzed.

If the network is directed, for each node 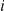 we define the (total) degree as 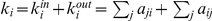, whereas 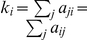 for undirected network. The average degree is given by 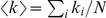. Similarly, for a directed network the in-, out-, and total strength of node 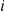 are given by 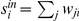, 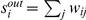, and 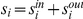, respectively, and the total network weight by 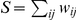. If the network is undirected we have instead 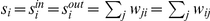 and 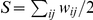.

A 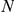-state Markov chain 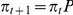, with 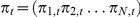, can be associated to the 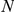-node network by row-normalizing the weight matrix 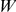, namely by letting the transition probability from 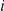 to 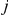 equal to



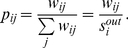
(1)


The quantity 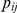 is the probability that a random walker which is in node 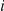 jumps to node 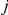, and 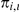 is the probability of being in node 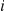 at time 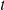. The transition matrix 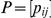 is a row-stochastic (or Markov) matrix (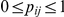 for all 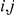, and 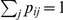 for all 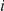). Furthermore, 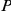 is irreducible since the network is connected. This implies that the equation 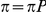 has a unique solution 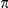, which is strictly positive (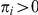 for all 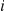) [23] and corresponds to the stationary Markov chain state probability distribution. For undirected networks one can easily check that 
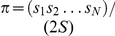
, whereas for directed networks a general closed form does not exist and 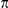 has to be numerically computed.

We denote by 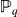 a partition of 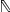 in 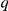 subsets (or subnetworks), namely 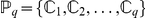 with 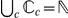 and 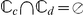 for all 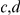. The sub-network 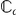 is called a (candidate) *community* (or *cluster*). Defining a partition 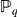 induces a 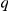-state meta-network, where communities become meta-nodes. The rigorous description of the dynamics of the random walker at this scale by a *lumped Markov chain*, however, is not possible if not in special cases [24] - actually, the Markovian property is not even preserved in general. Despite this limitation, a 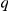-state Markov chain can be defined, which correctly describes the random walker at the aggregate level provided the stochastic process is started at the stationary distribution 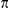 [25], [26]. This lumped Markov chain is defined by the 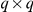 row-stochastic matrix



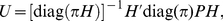
(2)where 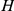 (*collecting matrix*) is a 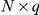 binary matrix coding the partition 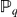, i.e., its entry 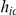 is 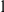 if and only if node 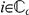 (see the Supporting Information S1 for the derivation of equation (2)). The lumped Markov chain 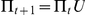 shares the stationary distribution with the original one (suitably collected), namely 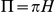 satisfies 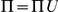. On the contrary, starting from an arbitrary 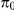, the lumped Markov chain 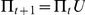 started at 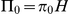 provides, in general, only an approximate description of the evolution of 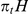. The difference between the real and approximate 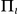, however, tends exponentially to zero if the two chains are regular [23], since they converge, by definition, to the same stationary state.

The ability of the lumped Markov chain to describe the random walk dynamics only at stationarity is not a limitation for our purposes, as it will be demonstrated by the examples of application. Note that the entry 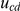 of 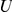 is the probability that the random walker is at time 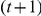 in any of the nodes of community 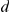, provided it is at time 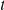 in any of the nodes of community 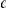. We define *persistence probability* of community 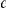 the diagonal term 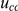. Large values of 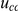 are expected for meaningful communities. In fact, the expected escape time from 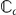 is 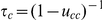: the walker will spend long time within the same community if the weights of the internal edges are comparatively large with respect to those pointing outside. Given a value 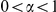, 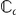 is defined 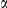-*community* if 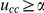. Thus 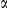 acts as a selection parameter, as sharply qualifies communities with respect to a given quality threshold. Consistently, 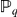 is defined 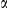-*partition* if it is composed of 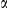-communities, namely 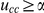 for all 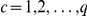.

### Testing communities

Testing the quality of a given partition is the simplest use of persistence probabilities. The partition can be the outcome of a community detection method (e.g., max-modularity) or instead derive from some *a priori* division (e.g., countries of the same continent in a financial network, or students of the same class in a school). By computing the 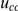-s using equation (2), the quality of each community and of the entire partition is readily quantified.

**Figure 1:**
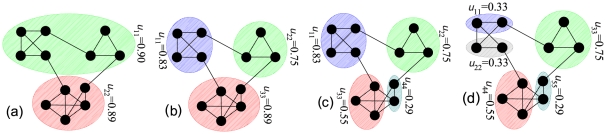
Four different partitions (with increasing number 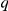
 **of communities) of the same network.** The persistence probabilities 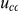 remain large as long as the network is partitioned into “natural” communities, or unions of them. Passing from (b) to (c), and from (c) to (d), significant communities are broken, with a sudden drop of the relevant persistence probabilities.

Consider the simple 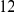-node network of Fig. 1 [27], which is purposely composed of three clusters. Four partitions are considered, corresponding to finer and finer divisions, and the 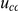-s are computed for each candidate community. As long as the communities coincide with “natural” clusters, or with the union of two of them, all the 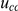-s remain rather large. But as soon as a natural community is broken, some very low persistence probabilities are found. If, for example, the quality level 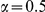 is fixed (a value having an important interpretation - see below), only the first and second partition are such that 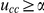 for all 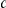 (i.e., they are 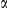-partitions). But even if the third and fourth partition fail in meeting such a requirement, yet some of their clusters can individually be classified as 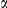-communities.

From equation (2), one can derive the explicit expression of the persistence probability 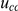 of cluster 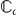 (see also the Supporting Information S1):



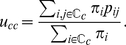
(3)


Kim et al. [28] note that 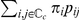 is the fraction of time that the random walker spends *on the links* internal to 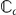. Thus 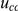 is the ratio between the latter and the fraction of time spent *on the nodes* of 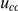. In the case of *undirected* network, recalling that 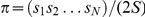, we obtain



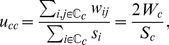
(4)having denoted by 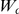 the *total internal weight* and by 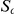 the *total strength* of 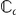. Thus the persistence probability has, in this case, a straightforward interpretation: it is the fraction of the strength of the nodes of 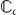 that remains within 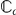.

In the even more special case of *unweighed* networks, this has a strict relationship, in turn, with the notion of “community in a weak sense” put forward by Radicchi et al. [14], who defined a community as a set 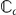 of nodes whose edges directed within 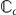 are more than those directed toward the rest of the network. It can easily be verified that this corresponds to 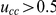. Therefore persistence probabilities generalize the above notion of “community in a weak sense” in a twofold direction: first, they extend it to weighted, directed networks; second, they allow a flexible tuning of the “strength” of the communities by fixing the desired minimum acceptable value (not necessarily 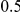) for 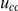.

We note that (again by restricting the attention to undirected, unweighed networks), it can easily be checked that 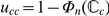, where 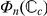 is the *normalized cut* of cluster 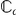 (e.g., [12] p. 92), namely the ratio between the number of edges connecting 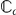 to the rest of the network and the sum of the degrees of the nodes of 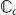. This observation bridges our dynamical, Markov-chain-based method with traditional graph partitioning techniques. It has already been pointed out that the latter are scarcely suitable for community detection [5], [12], because the number of clusters has typically to be provided *a priori* whereas, in most instances, it is part of the outcome the network analyst is seeking for (see [29] for a relationship between modularity and cut size). Nonetheless, in the next section we shall see how a flexible exploitation of persistence probabilities enables an effective community analysis.

### Finding communities

In the previous section, the persistence probabilities were used for *testing* given partitions and, individually, their communities. Here, instead, we want to analyze how this tool can be exploited for *finding* communities, namely for deriving partitions composed of meaningful communities.

The starting point is to define the desired level for the quality parameter 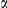. For example, as pointed out above, in the case of undirected, unweighed networks, the constraint 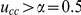 for all 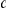 is equivalent to require partitions composed of “communities in a weak sense”, according to the definition of [14]. But the network analyst can be more or less restrictive, i.e., require a larger (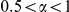) or smaller (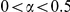) significance level.

In general, for any given 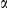, a large set of 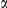-partitions exist, i.e., such that 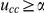 for all 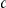 (e.g., the trivial partition 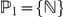, the entire network, is an 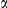-partition for any given 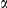). Typically one searches for small (yet significant) communities, to effectively decompose the network. Thus we can rigorously formulate the problem of community detection as follows:



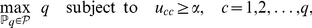
(5)where 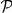 denotes the set of all partitions. Notice that the admissible set of problem (5) is not empty for any given 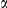 (since 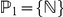 has 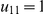) and that, in general, the optimal solution is not unique (if 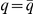 attains the maximum in (5), there can be many 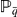 which are 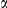-partitions).

Analyzing the theoretical properties of problem (5) is beyond the scope of this work (see [30] for a discussion on the NP-completeness of some related optimization problems). Instead, a heuristic approach for finding a suboptimal solution to (5) can readily be derived, by restricting the optimization to a (much smaller) subset 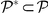 obtained by whatever “partitions generator”, namely an algorithm that yields a set of partitions 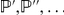 which are hopefully “good” candidates for community detection. In this way, problem (5) is readily solved by picking up the 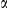-partitions within 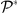, and taking the one(s) with the largest 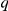. We will make reference to this procedure in the remainder of the paper, but we anticipate that, instead of the “unsupervised” approach just outlined (with 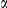 fixed *a priori*), we will often prefer a “supervised” approach consisting in first generating a bunch of meaningful partitions, then comparatively assessing their quality, and finally selecting the preferred one, thus implicitly fixing the 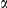 value *a posteriori*. We will illustrate this procedure through many examples.

Several methods have been proposed to derive network partitions which are meaningful in the sense of community analysis (see again [12] for a thorough analysis). All of them can be used in our framework: here we adopt a method for deriving partitions which is based on cluster analysis and is consistent with the above introduced random walk modeling.

Cluster analysis can be used to group “similar nodes” into candidate communities. This needs defining a meaningful *similarity/distance* among each pair of nodes. Such a definition is by no means obvious: among the many proposals [12], a few exploit random walks to induce a suitable similarity measure (e.g., [31]–[35]). We follow this line by proposing an approach in which, however, we do not explicitly perform random walks in a Monte Carlo fashion, but derive analytically the global behavior of a large number 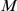 of walkers (a “fleet”) started from each node 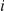.

Consider a large number 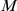 of repetitions of a random walk started from 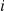. For each repetition, the probability that the walker is in 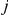 after 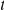 steps is 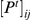. Thus, if 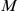 random walks of length 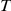 are performed from 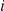, the expected number of visits to 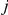 in any time instant in 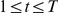 is 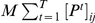. By averaging with respect to 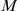, we propose a (symmetric) similarity 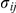 defined by



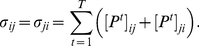
(6)


Note that this is conceptually equivalent to an explicit random walk approach, but with an arbitrarily large number 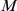 of repetitions from each starting node instead of one only. Most notably, the results do not depend on the actual stochastic realization of the random walks. We finally define the distance 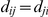 between nodes 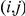 by complementing the similarity and normalizing the results between 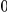 and 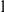:



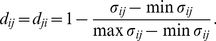
(7)


The rationale underlying the definition of 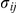 and 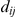 is to assign nodes 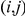 a large similarity if a numerous fleet of random walkers started in 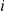 (resp. 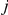) makes a large number of visits to 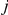 (resp. 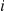) within a sufficiently small time horizon 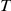. The notion of community induced by this metric, therefore, is that of a subnetwork where a random walker has a large probability of circulating for quite a long time, before eventually leaving to reach another group. This is conceptually consistent with the definition of 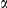-community above introduced.

The choice of the time horizon 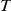 is potentially critical. Cluster analysis yields a different hierarchical tree (*dendrogram*) for each time horizon 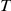, whose choice is thus nontrivial. At the two extremes, setting 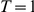 restricts the pairs of nodes which are candidate to nonzero similarity to neighboring pairs only, whereas larger and larger values of 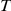 tend to make any node equally similar to any other. We found that an effective selection of 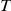 can be empirically obtained by maximizing the *cophenetic correlation coefficient* 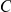, which is defined as the linear correlation between the distances 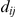 and the *cophenetic distances* 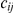 [36]. The latter are a product of the hierarchical cluster analysis: for any node pair 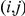, the cophenetic distance 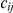 is the height of the link joining (directly or indirectly) nodes 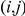 in the dendrogram. The value of 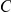 is generally used to assess whether the adopted distance 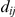 induces an effective clusterization (notice that 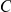 qualifies the entire dendrogram, and not a network partition), although limitations have been observed in specific applications [37].

**Figure 2:**
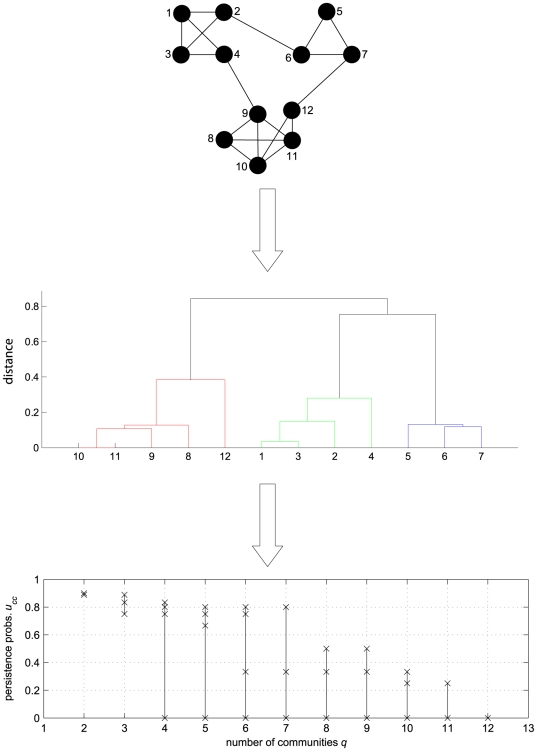
Summary of the procedure for community analysis. From the network description (top panel) and a suitable definition of node distance, a hierarchical tree (dendrogram) is derived by cluster analysis (middle panel). Horizontal top-down cross-sections of the dendrogram identify a sequence 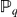 of partitions with increasing number 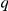 of candidate communities. In the *persistence probabilities' diagram* (bottom panel), the 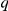 diagonal terms 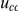 of the lumped Markov matrix 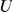 are plotted for each partition 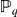 (crosses denote the values of the 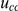, vertical straight lines are only for visual aid). In this example, the sudden drop of the least 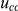 for 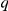 larger than 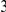 reveals that a meaningful community has been broken passing from 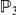 to 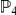.

The entire procedure for finding communities is summarized in Fig. 2 with reference to the toy-network of Fig. 1. Starting from the network description, we apply cluster analysis for each 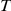 ranging from 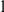 to some sufficiently large 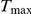 (of the order of 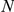), eventually taking the 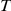 value that maximizes 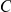. Horizontal top-down cross-sections of the associated dendrogram identify a sequence 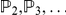 of partitions with increasing number 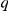 of candidate communities. For each 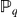 we compute the lumped Markov matrix 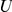 according to (2), and plot its diagonal terms in the *persistence probabilities' diagram*. In the case of Fig. 2, the sudden drop of the least 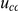 for 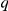 larger than 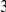 reveals that a meaningful community has been broken passing from 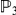 to 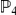. If we set, for instance, the quality threshold at 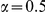, then 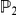 and 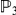 can be qualified as 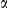-partitions, and thus 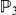 will be our choice if we seek for the finest partition, consistently with problem (5).

## Results

The analysis of four networks is now discussed. We will consider two families of synthetical benchmark networks with built-in cluster structure; a real-world network with a rather strong community structure; and another real-world network with weak clustering but with a few well-defined communities. Other examples are discussed in the Supporting Information S1.

### LFR benchmarks

Lancichinetti, Fortunato, and Radicchi (LFR) [38] proposed a family of synthetically generated graphs, designed to serve as benchmarks for testing community detection algorithms. They explicitly took into account two properties found in real networks, namely the heterogeneity in the distributions of node degrees and community sizes. Both of the latter are taken as power laws, with given exponents 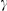 and 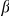, respectively. In addition, the network is defined by prescribing the number 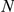 of nodes, the average degree 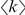, and a *mixing parameter* 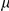 such that each node shares a fraction 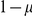 of its edges with the other nodes of its own community, and a fraction 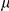 with the rest of the network. The benchmark generating method was later extended to oriented and weighted networks [39] (see the Supporting Information S1) - here we consider undirected, unweighed networks with 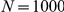, 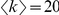, 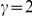. We first let 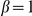 and 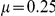. Since the generating algorithm is stochastic, we produce 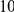 different network instances: the number of built-in communities 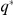 turns out to range from 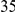 to 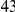, and the size of each community from 10 to 77 nodes.

**Figure 3:**
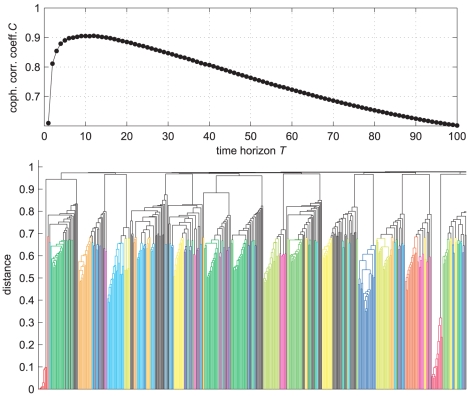
LFR benchmark networks. Above: The cophenetic correlation coefficient 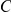 as a function of 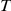 for one of the network instances. The maximum is attained at 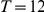. Below: The dendrogram obtained with 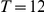 (only half of the plot is shown for readability).

We now fix our desired quality level, for example 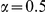, and solve problem (5) for each of the 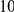 networks. For that, we use the above described “partitions generator”: in Fig. 3 we show, for illustrative purposes, the cophenetic correlation coefficient 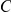 as a function of the random walk time horizon 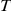, as obtained analyzing one of the networks. We find a unimodal dependence, as for almost all the network studied. We take therefore 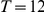 in this case, which attains the maximum 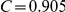. The related dendrogram is in the same figure.

**Figure 4:**
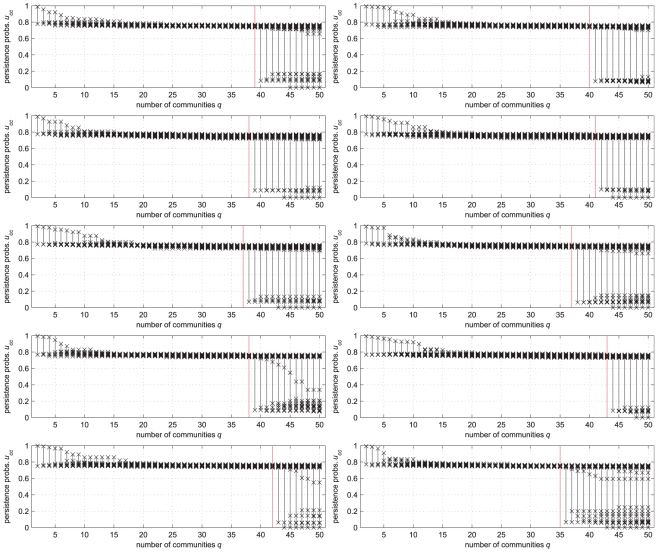
The persistence probabilities' diagrams of the LFR benchmark networks with 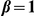
 **,** 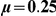 **.** (See the text for the other parameters). The vertical red line marks the built-in number of communities. In all instances, this value is revealed by a sudden drop of some of the persistence probabilities.

The persistence probabilities' diagrams obtained for the 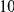 networks are shown in Fig. 4. In all instances, the diagrams reveal a sharp discontinuity. For 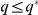, all the 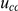-s are rather large (larger than 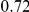). This indicates that meaningful communities are identified. For 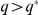, instead, some significant communities are broken, as revealed by a larger and larger number of small 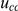-s. In other words, the correct number of built-in communities is systematically revealed, in all instances, by a sudden drop of some of the persistence probabilities. This implies, in turn, that solving problem (5), i.e., taking the largest 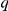 such that 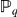 is an 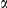-partition with 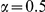, yields a solution with 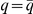 which exactly recovers the number 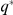 of communities. Furthermore, such a solution is largely insensitive to the choice of the quality level: for example, any value in a range 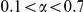 would give the same result.

Obviously, the fact that 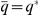 does not imply that the two partitions are identical. In order to quantify the ability of the method, we compare the built-in partition with that obtained by solving problem (5), in terms of the *normalized mutual information* 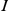, a reliable and often used measure of partition similarity introduced by [40] to the network research community. The definition of 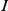 is reported in the Supporting Information S1: here we only point out that 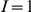 when the two partitions are identical, whereas 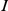 has zero expected value for independent partitions. We obtain an average of 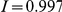 over the 10 networks, which favorably compares to the values reported by [38] after extensive tests by using modularity optimization (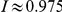) and Potts model clustering [41] (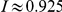).

In [38] it is shown that the performance of community detection algorithms deteriorates when, *ceteris paribus*, the scale parameter 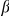 of the power-law community size distribution increases (i.e., communities are less differentiated in size) and/or when the mixing parameter 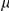 increases (i.e., communities become less isolated each other). To analyze this situation, we generate another set of 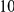 benchmark networks by increasing 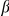 from 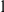 to 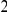 and 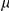 from 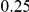 to 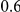 (the highest 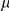 value considered in [38]): the resulting networks turn out to have from 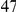 to 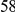 communities, with size ranging from 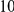 to 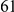 nodes. Notice that we are generating low-quality clusters, due to the large 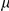: actually, none of them would met the requirement of “community in a weak sense” according to [14]. In other words, the cluster structure of the network is extremely weak, and that is obviously the reason of the scarce performance of community detection tools.

**Figure 5:**
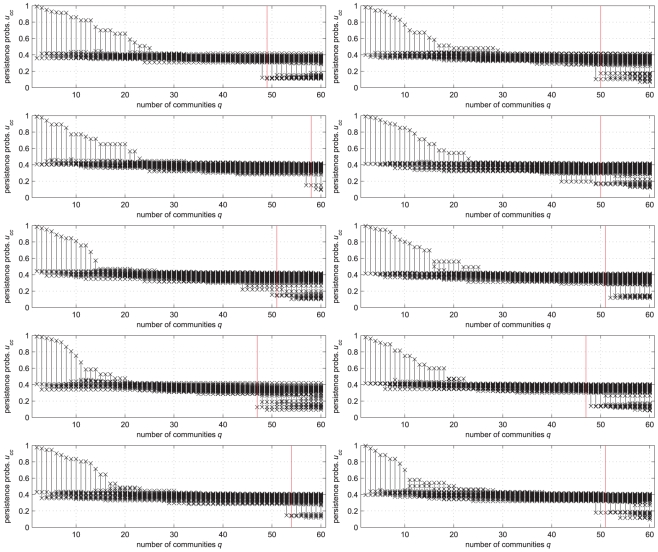
The persistence probabilities' diagrams of the LFR benchmark networks with 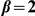
 **,** 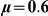 **.** (See the text for the other parameters). The vertical red line marks the built-in number of communities. The persistence probabilities accumulate around 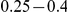, denoting the low quality of the clusters (compare with Fig. 4).

All of this is captured by the persistence probabilities' diagrams of Fig. 5. All the candidate partitions are characterized by low-quality clusters (with the 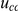-s accumulating in the range 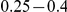), which is the obvious result of the low quality of the built-in partitions. In this situation, when analyzing one of the networks, the detection procedure of [14], based on the notion of “community in a weak sense”, would discard any candidate community; the max-modularity approach would yield a partition as outcome, but with no assessment of the quality of its clusters; and also the unsupervised solution of our problem (5) would lead to poor results: for example, setting *a priori* the value of 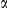 to the “standard” value of 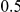 would discard all partitions.

It is exactly in such a difficult context that persistence probabilities can be a precious decision support tool. By looking at the diagrams of Fig. 5, the analyst immediately grasp the weak cluster structure of the network under scrutiny, and can consistently *a posteriori* fix an 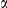 value not unrealistically restrictive. Alternatively, he/she can rely on the observation of a sudden drop in one or more persistence probabilities' as an indication of a (comparatively) good partition. This means selecting 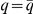 such that 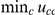 has the largest variation from 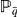 to 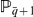. If we systematically apply this strategy to the 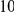 benchmark networks, we obtain an average mutual information between the built-in and the obtained partition of 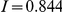, which is intermediate with respect to the values obtained by [38] with modularity optimization (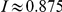) and Potts model clustering (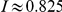). But the added value of our approach is, for the selected partition 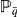, the quality measure 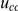 of each cluster and, consequently, of the entire partition.

### Netscience network

The Netscience network is a weighted, undirected, social network describing the collaborations (up to year 2006) among researchers in network science, the weight of the edge connecting two researchers being proportional to the number of papers they have co-authored [18]. Its giant component has 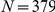 nodes, and it is generally considered an example of a real network with a rather strong community structure. Many methods for network analysis, included community detection algorithms, have been tested and discussed on this example (e.g., [42]-[44]).

**Figure 6:**
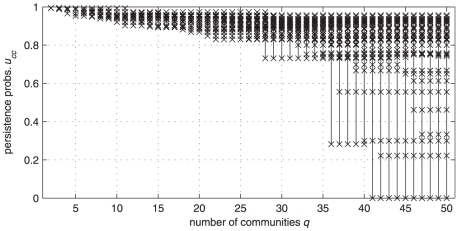
The persistence probabilities' diagram of the Netscience network.

If we run our partitions generator algorithm, at 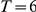 we get the dendrogram attaining the largest 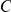: the resulting persistence probabilities' diagram is in Fig. 6. The plot has a less sharp structure than that of the LFR networks of Fig. 4: if we adopt once again the criterion of [14], namely we solve problem (5) in an unsupervised fashion by letting 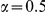, then 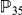 is the optimal partition (here we have straightforwardly extended the notion of “community in a weak sense” to weighted networks). In a supervised approach, instead, the network analyst will select the proper 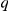 as a trade-off between a finer decomposition (large 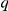) and a higher significance of the communities (small 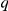). For example, setting 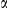 as large as 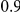 yields 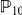 as the optimal partition, i.e., the 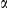-partition with largest 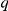.

It is instructive to compare these results with those obtained, on the same case study, by the *graph stability* approach proposed by Delvenne et al. [42] (a detailed comparison of the two methods is in the next section). By means of the KVV algorithm [45] (a hierarchical, divisive, non-binary, graph clustering method), they obtain a sequence of six partitions, with 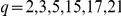. Analyzing and comparing the *stability curve* (i.e., the autocovariance function of a signal emitted by a random walker) of each of them, the authors suggest their partition with 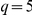 as the more reliable, as it has the largest stability over a longer time span with respect to any other. Incidentally, this is also a supervised approach that leaves the analyst the choice of the preferred solution among a set of alternatives.

**Figure 7:**
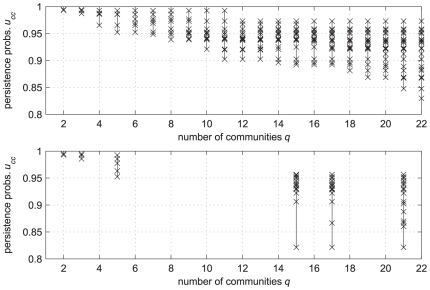
Comparison of two persistence probabilities' diagrams for the Netscience network. The two plots are in the same scale. Above: blow-up of the diagram of Fig. 6 (our results). Below: the diagram related to the six partitions proposed in [42].

In order to test the six partitions of [42], we created their persistence probabilities' diagram and compared it with our results in the diagram of Fig. 7. The partition 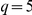 of [42] confirms to be definitely more significant than those with finer decomposition (i.e., 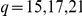) according to the criterion of minimal 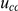 too. Actually, our and their 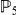 partitions share the same minimal 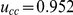, due to a common 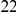-node community. They are, however, partially different (their normalized mutual information is 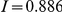, with about 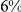 of differently classified node pairs).

The inspection of Fig. 7 also reveals that, for each given 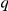, the partitions obtained with our method are superior than those proposed in [42], provided the criterion put forward in this paper (i.e., minimal 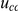) is adopted. Actually, while the criterion of [42] ranks partitions by “averaging” among the communities, our approach is a “worst-case” one: by selecting an 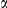-partition one guarantees that the “worst” community has a persistence probability not less than 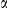. Finally, note that in the gap from 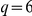 to 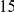, where no partition is obtained by the KVV divisive algorithm, our partitions generating algorithm provides a set of finer and finer partitions, whose quality only slowly deteriorates as 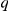 increases. The network analyst can fruitfully select in this interval a proper trade-off between fine granularity and significance of the partition.

### World trade network

The final example concerns a real-world, directed, weighted network, representing the trade flows among countries. This network, denoted as *world trade network* (or *world trade web*), has extensively been studied in recent years (e.g., [46]–[48]). The problem of the existence of communities, namely groups of countries with preferential partnerships, has been addressed too, although results seem to be not definitive [49], [50]. This issue is obviously related to the debate about “globalization versus regionalization” in the world economy.

**Figure 8:**
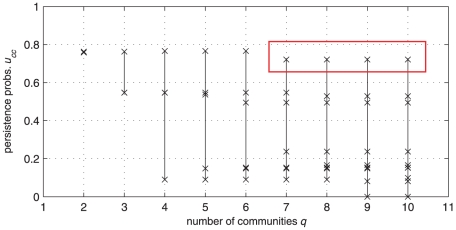
The persistence probabilities' diagram of the world trade network. Overall, the network does not display a definite community structure. However, a single cluster with rather strong internal connectivity (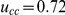) is detected (as evidenced by the rectangle), corresponding to a subnetwork which includes almost all European countries.

We consider the network derived from 2008 data, whose largest connected component has 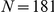 nodes. It does not seem to display a definite community structure: as a matter of fact, the maximum modularity (estimated as in [51]) is rather small, namely 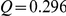, if compared to other examples where 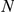 has the same order of magnitude (e.g., 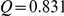 for the Netscience network). In this situation, we show how our method is able to detect well-defined communities (if any) even in a network which overall does not possess a definite clusterized structure. Consider the persistence probabilities' diagram of Fig. 8. With the exception of the cases 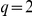 and 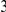, corresponding to rather trivial partitions, no 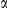-partition exists with 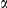 reasonably large (say, 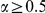). Nonetheless, an 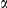-community which meets a restrictive quality standard is stably detected in a rather wide range of 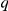, as highlighted in the figure. It is a cluster composed of 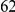 nodes which shows a rather strong internal connectivity (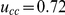). Any other candidate cluster, instead, turns out to have a much smaller 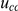 value and, therefore, it can hardly be considered to be a significant community. Interestingly, this meaningful cluster includes almost all European countries, plus a number of minor non-European partners.

## Discussion

### Comparison with other community detection methods

In the section “Methods” we highlighted some important connections between persistence probabilities and other quantities which are standard in graph theory. As we pointed out, for undirected networks 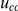 reduces to the so-called *internal density*, namely the ratio between the total internal weight and the total strength of 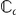. In turn, if the graph is unweighed too, this turns out to be one minus the normalized cut of 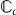. The definition of “community in the weak sense”, put forward in [14], can also be reinterpreted in terms of persistence probabilities. No straightforward connections, however, can be deducted for directed networks, where nonetheless the tool of persistence probabilities can be fully applied.

An important relationship between random walks and modularity is put forward by Kim et al. [28] who propose their *LinkRank modularity* 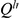 (that we denote by 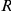 for clarity), a variation to the standard modularity aimed at obtaining a better performance on directed graphs. In words, 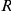 is the difference between the fraction of time spent walking within communities (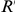) and the expected value of this fraction on a suitable null model (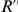). Both these terms are additive with respect to communities, and it turns out that (with our notation):



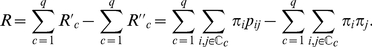
(8)


In the case of undirected networks, simple computations show that 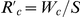 and 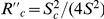, which implies that the LinkRank modularity reduces to the standard one 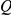, which indeed can be written as:



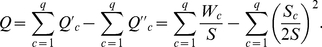
(9)


The comparison between 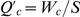 and the persistence probability 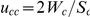 reveals obvious analogies but also subtle and important differences. The former is the fraction of time spent in community 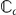: being proportional to the total internal weight 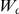, it will be smaller for smaller clusters, *ceteris paribus*, regardless to their cohesiveness. On the contrary, 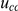 measures the probability of remaining in 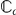 given that the walker is currently there, regardless to the dimension of the cluster, thanks to the normalization by the total cluster strength 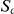. The result is a superior capability of persistence probabilities is assessing the quality of clusters whatever their size is, a precious feature when analyzing multi-scale networks (i.e., composed of communities of different size scales).

**Figure 9:**
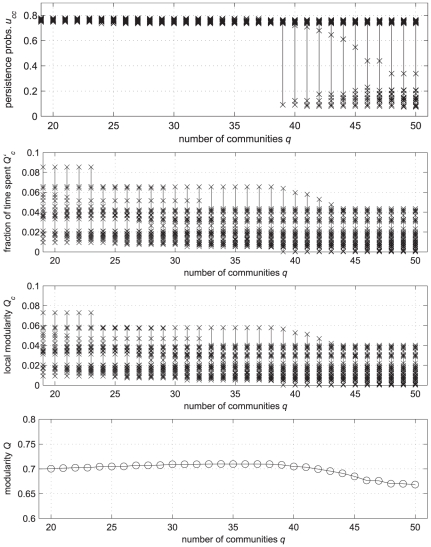
A comparison between persistence probabilities and fraction of time spent as indicators of the quality of a community. The test considers a LFR benchmark network with 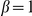, 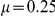 (see the text for the other parameters). For each candidate partitions 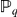, the four panels show, from the top to the bottom: the persistence probabilities 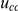; the fractions of time 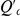 spent in each community by a random walker; the difference of such fractions with those obtained in a null network model (local modularity 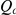); the modularity 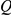 of the partition. Only the set of persistence probabilities shows a definite structural change in correspondence of the correct number of communities (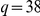).

This can be demonstrated, for example, by considering an instance of a LFR benchmark network with 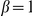, 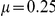 (see the section “Results” for the value of the other parameters) and analyzing the values of 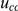 and 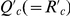 for a set of partitions. The network has 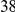 communities with size ranging from 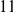 to 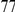 nodes. We use our partitions generator to yield a family of 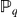 with 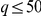. For each partition, we compute the set of persistence probabilities 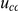 and the set of the fractions of time spent in the community 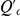. The results are shown in the first and second panel of Fig. 9: as long as the considered 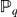 does not break any of the built-in clusters, all the 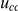-s remain large and concentrated in a rather narrow range, regardless to the cluster size. Then, some of them abruptly decreases as soon as clusters are broken. The 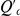-s, on the contrary, are quite widespread (in a range from 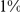 to 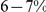) and vary in a rather smooth manner, since a smooth reduction of cluster sizes yields a corresponding smooth reduction of their internal weight 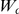. It seems therefore that the fraction of time spent walking is not as indicative of the quality of a cluster as the persistence probability. The scenario does not modify if the *relative* fraction of time 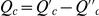 (or *local modularity*) is considered, i.e., if the comparison with the performance of a null model is accounted for, as it appears from the third panel of Fig. 9. The obvious consequence is a very small sensitivity of the modularity 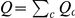, at least within this set of partitions. As shown in the bottom panel of Fig. 9, 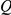 has almost the same value in 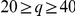, which makes questionable the reliability of choosing the max-modularity partition (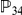, in this case). Further discussion on the use of absolute vs. relative (i.e., compared with a null model) cluster measures is in the Supporting Information S1.

The proposed approach has also important connections with two recently published community analysis methods. Delvenne et al. [42] show that the autocorrelation function of a signal emitted by a random walker, with value 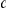 as long as the walker is in a node 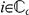, can be expressed in terms of the clustered autocovariance matrix 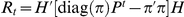, and they define the stability of the partition 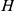 as 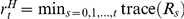. Given a set of candidate partitions, the *graph stability* function 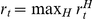 puts in evidence, for each time instant 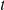, which is the “optimal” partition. It is suggested in [42] that the most relevant partitions are those which are optimal over long time windows. It is straightforward to check that our matrix 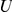 is related to the step-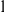 autocovariance 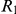 by 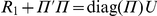. The two methods are thus based on the same ground, but our approach has two advantages: first, for each partition 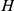 we do not have to compute a long time-dependent sequence such as 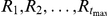 (with 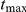 of the same order as 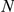) of 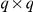 matrices, but the sole matrix 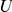, with an important reduction in the computational burden. Second, the full list of the persistence probabilities 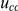 allows one to test the quality of each single community, whereas the stability of the clustering 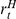 averages among all the communities.

Finally, a work with straightforward connections to ours is that of Weinan et al. [52], who suggest that the best 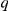-community partition is that corresponding to the “best” (in a suitable technical sense) 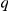-state approximated lumped Markov chain. This boils out to the formulation of a minimization problem, after a metric on the space of stochastic matrices is introduced. A drawback of this approach is however that 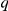 must be *a priori* specified, whereas often identifying the correct number of communities is the main goal of the analysis. For the same reason, it can hardly support the discussion of the significance and convenience of choosing one partition instead of another.

### Concluding remarks

In this paper, we have shown that associating a lumped Markov chain to a given network partition (i.e., a set of communities) provides an effective tool for testing the significance of each single community and, consequently, of the entire partition. As a matter of fact, the diagonal terms (called persistence probabilities) of the lumped Markov matrix can be used as quality measures for each individual community. If a threshold level 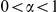 is fixed, a sharp criterion for defining a community as “meaningful” is therefore that of requiring that its persistence probability is not less than 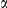.

If an effective method for generating a set of “good” partitions is available, the above criterion can be used to rapidly select one of them among those complying with the prescribed 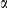-quality, typically the one with the finest network decomposition (i.e., the largest number of communities). We have used a generator of partitions based on hierarchical cluster analysis, where the node distance is again defined on the basis of a Markov chain random walk model. Overall, the method has fair computational requirements, and can be applied to fully general networks (i.e., directed and weighted). Its effectiveness has been demonstrated on several medium-scale examples (see also the Supporting Information S1 for further case studies).

As already pointed out, the tool of persistence probabilities can be used to assess the quality of partitions, or single clusters, obtained with whatever method (e.g., modularity optimization) or *a priori* defined (e.g., geographical areas in the world trade network). Along this line, two possible extensions appear to be promising. One one side, several methods have recently been proposed to identify *overlapping* communities, i.e., clusters with shared nodes [53], [54]. In principle, a lumped Markov chain can be associated to a *cover* as well (i.e., a clusterization with possible overlaps), although this requires a careful treatment of the shared nodes. Another extension concerns time-variant networks, namely networks whose edges (or their weights) vary in time (many examples can be found in social or economic networks). Once a community structure has been identified in a given time instant (i.e., on a “frozen” network), one may be interested in tracking the time evolution of the persistence probabilities, to reveal which communities remain significant in time or, on the contrary, which ones have a decaying cohesion [22], [55]. These extensions will be the subject of future research.

## Supporting Information

Supporting Information S1
**Figure S1.1.** The persistence probabilities' diagram of the Erdös-Rényi network. **Figure S1.2.** Zachary's karate club network. Above: The dendrogram obtained with 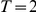. Below: The persistence probabilities' diagram. **Figure S1.3.** The persistence probabilities' diagrams of two LFR directed, weighted benchmark networks. Top: 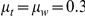 (the number of planted communities is 35). Bottom: 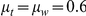 (42 planted communities). See the text for the other parameters. **Figure S1.4.** LinkRank benchmark network. **Figure S1.5.** The persistence probabilities' diagram of the LinkRank benchmark network. **Figure S1.6.** The persistence probabilities' diagram of the neural network. **Figure S1.7.** Absolute and relative persistence probabilities' diagrams of a LFR benchmark network. The relative persistence probability 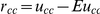 compares the absolute one 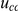 with the persistence probability 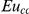 of the same cluster in a null model.(PDF)
