# Supplementary material for: Finding and Testing Network Communities by Lumped Markov Chains
Source: PLoS One. 2011 Nov 3;6(11):e27028. doi: 10.1371/journal.pone.0027028 (PMC3207820; doi:10.1371/journal.pone.0027028)
Supplement: Supporting Information S1 — Figure S1.1. The persistence probabilities' diagram of the Erdös-Rényi network. Figure S1.2. Zachary's karate club network. Above: The dendrogram obtained with . Below: The persistence probabilities' diagram. Figure S1.3. The persistence probabilities' diagrams of two LFR directed, weighted benchmark networks. Top: (the number of planted communities is 35). Bottom: (42 planted communities). See the text for the other parameters. Figure S1.4. LinkRank benchmark network. Figure S1.5. The persistence probabilities' diagram of the LinkRank benchmark network. Figure S1.6. The persistence probabilities' diagram of the neural network. Figure S1.7. Absolute and relative persistence probabilities' diagrams of a LFR benchmark network. The relative persistence probability compares the absolute one with the persistence probability of the same cluster in a null model. (PDF) [file pone.0027028.s001.pdf]

Supporting Information S1
Finding and Testing Network Communities by Lumped Markov Chains

Carlo Piccardi

Department of Electronics and Information, Politecnico di Milano, Milano, Italy

E-mail: carlo.piccardi@polimi.it

Derivation of the transition matrix of the lumped Markov chain

We use the notation introduced in the main text. First note that the entry (i,j) of $\Lambda = \text{diag}(\pi)P$, namely $[\Lambda]_{ij} = \pi_i p_{ij}$, is the joint probability that the random walker is in i at time t and in j at time $(t+1)$. Then, the collecting matrix H aggregates such probabilities within each community, that is, the entry (c,d) of $\Gamma = H'\text{diag}(\pi)PH$, namely

$$[\Gamma]_{cd} = \sum_{i\in\mathbb{C}_c}\sum_{j\in\mathbb{C}_d}\pi_i p_{ij}, \tag{1}$$

is the joint probability of being in any node of $\mathbb{C}_c$ at time t and in any node of $\mathbb{C}_d$ at time $(t+1)$. The required meta-network transition probabilities u_{cd} are finally obtained by dividing each row c of Γ by the stationary probability of being in $\mathbb{C}_c$, namely by $\theta_c = \sum_{i\in\mathbb{C}_c}\pi_i$. This is performed by left-multiplying Γ by $[\text{diag}(\pi H)]^{-1}$, since $\pi H = (\theta_1\theta_2\ldots\theta_q)$.

It is now straightforward to prove that the original Markov chain $\pi_{t+1} = \pi_t P$ and the lumped one $\Pi_{t+1} = \Pi_t U$ have the same stationary distribution (provided the latter is suitably collected), namely $\pi = \pi P$ implies that $\Pi = \pi H$ satisfies $\Pi = \Pi U$. For that, we have to evaluate $\Pi U = \pi HU = \pi H[\text{diag}(\pi H)]^{-1}H'\text{diag}(\pi)PH$. But $\Psi = \pi H[\text{diag}(\pi H)]^{-1} = (11\ldots 1)$ (a $1\times q$ vector), and $\Omega = H'\text{diag}(\pi)$ is a $q\times N$ matrix whose only nonzero entry of column i is π_i, which is located in row c if $i\in\mathbb{C}_c$. Thus $\Psi\Omega = \pi$, and hence $\Pi U = \pi HU = \pi PH = \pi H = \Pi$.

Normalized Mutual Information

Consider a network with N nodes and two partitions $\mathbb{P}'_R = \{\mathbb{C}_1, \mathbb{C}_2, \ldots, \mathbb{C}_R\}$ and $\mathbb{P}''_S = \{\mathbb{C}_1, \mathbb{C}_2, \ldots, \mathbb{C}_S\}$, with R and S communities, respectively. Denote by n_{ij} the number of nodes classified in $\mathbb{C}_i$ by partition $\mathbb{P}'_R$ and in $\mathbb{C}_j$ by partition $\mathbb{P}''_S$, and define also $n_i = \sum_j n_{ij}$ and $n_j = \sum_i n_{ij}$. Then the normalized mutual

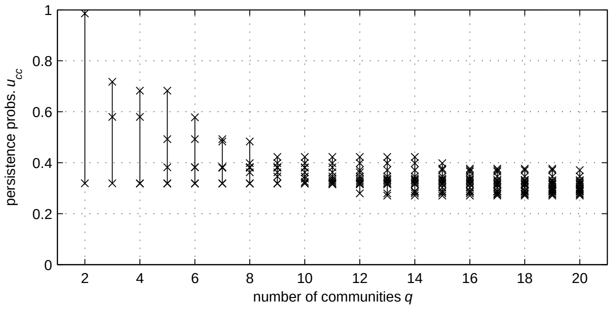

Figure S1.1. The persistence probabilities' diagram of the Erdős-Rényi network.

information between $\mathbb{P}'_R$ and $\mathbb{P}''_S$ is defined as

$$I = \frac{-2\sum_{i=1}^{R}\sum_{j=1}^{S} n_{ij}\log\left(\frac{n_{ij}N}{n_i n_j}\right)}{\sum_{i=1}^{R} n_i \log\left(\frac{n_i}{N}\right) + \sum_{j=1}^{S} n_j \log\left(\frac{n_j}{N}\right)}. \tag{2}$$

A discussion on the interpretation of I and on its main properties can be found in [1] (see also [2]).

Applications and Examples

We enlarge the scope of the results presented in the main text by illustrating a few more examples of application.

Erdős-Rényi network

By construction, Erdős-Rényi (ER) networks should not display communities, as they are built by randomly, homogeneously distributing edges among nodes (although exceptions can arise due to stochasticity [3]). We build a ER network with $N = 1000$ and $\langle k \rangle = 10$. The analysis confirms the lack of a clusterized structure: at the optimal time horizon $T = 4$ the cophenetic correlation coefficient C is as small as 0.437, and the persistence probabilities' diagram of Fig. S1.1 shows the absence of α-partitions with reasonably large α. Also, rather significant communities exist only for trivial (i.e., very small) q.

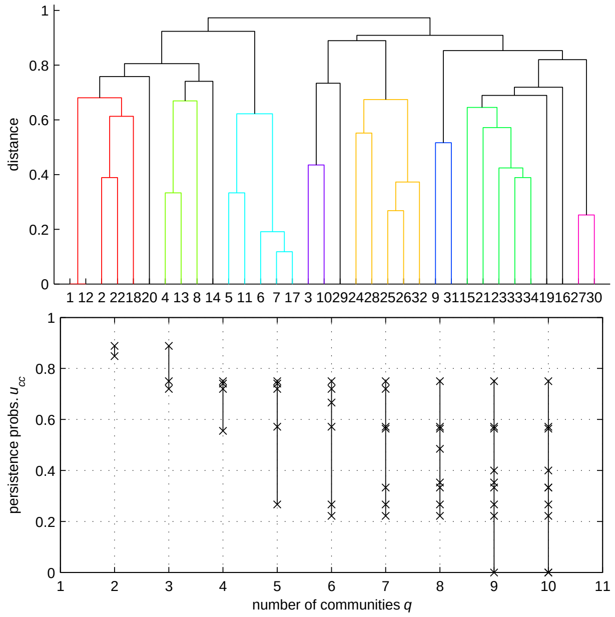

Figure S1.2. Zachary's karate club network. Above: The dendrogram obtained with $T = 2$. Below: The persistence probabilities' diagram.

Zachary's karate club

Zachary's karate club [4] is a 34-node network which has become a standard benchmark in community detection [2], despite its very small dimension. By applying our method, we obtain at $T = 2$ (for which C is maximal) the dendrogram and the persistence probabilities' diagram of Fig. S1.2. As usual, the quality of the partitions deteriorates as q increases: setting the standard value $\alpha = 0.5$ yields $\mathbb{P}_4$ as the optimal solution of the community detection problem (problem (5) in the main text): incidentally, this is also the max-modularity partition. More restrictive α values lead instead to $q = 2$ or 3.

It is interesting to note that the "historical" 2-way partition, as reported by Zachary [4], has $\mathrm{diag}(U) = (0.875, 0.868)$, and that we found the largest $\min u_{cc} = 0.872$ with the 2-way partition obtained by Weinan et al. [5]. We exactly recover the same $\mathbb{P}_2$ partition with our partitions generator if we let the time horizon $T = 1$ instead of 2. This suggests that the performance of our algorithm can in principle be improved if one performs a fine tuning of T. This can easily be done for small-size networks, but can be problematic for large ones.

LFR benchmark

LFR benchmark networks [6] have already been introduced in the main text. Since they are nowadays the most important and structured set of benchmarks for community detection algorithms, we test our method on some other sample network. More specifically, we move to directed, weighted networks, which can be generated by an extension of the basic LFR procedure, as described in [7].

Figure S1.3 displays the results obtained with two networks. Their parameters (we refer to [7] for their detailed definition) are $N = 1000$, $\langle k \rangle = 25$, $\tau_1 = -2$ and $\tau_2 = -1$ (exponents of the power-law degree and community size distributions, respectively), $\beta = 1.5$ (coefficient of the degree-weight relationship). The two networks differentiate for the topology and weight mixing parameters, which are set to $\mu_t = \mu_w = 0.3$ and $\mu_t = \mu_w = 0.6$, respectively. The results are qualitatively similar to the undirected, unweighed case (see the main text): in both instances the correct number of planted communities is perfectly identified by a sharp decrease in the minimal u_{cc}. By the way, the 35 communities are perfectly identified in the first case (with a normalized mutual information $I = 1$) and almost perfectly in the second one ($I = 0.9996$). Furthermore, the different quality of the communities in the second case, due to the larger mixing, is revealed by the smaller u_{cc} values.

LinkRank benchmark

Kim, Son, and Jeong [8] proposed a variation to the standard modularity, in order to improve the ability of the method in the specific case of directed networks. Their proposal is based on the introduction of a quantity called LinkRank, which is a measure of the importance of an edge (see the main text for a thorough discussion). They demonstrate the superiority of their approach on the benchmark graph of Fig. S1.4, which is composed of 8 rings of 8 nodes each (thus $N = 64$). Rings are intended to form communities, as a walker can circulate indefinitely within each of them. Furthermore, each ring is connected to another ring only by a single edge, forming a giant loop. Whereas the weights of the intra-ring edges are all set to $w = 1$, a large weight of the inter-ring edges (approximately above $w = 4$) can confound community detection algorithms [8]. We set $w = 5$ for the inter-ring edges, to test whether our approach is able to cope with this problem.

The results of the analysis are in Fig. S1.5. The correct community structure is perfectly identified, as it can be checked by inspecting the partition with $q = 8$. Actually, taking $q = 8$ gives rise to a 8×8

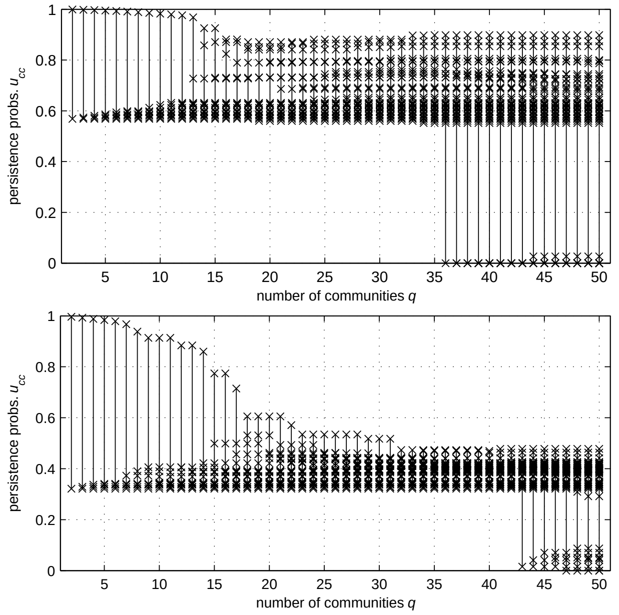

Figure S1.3. The persistence probabilities' diagrams of two LFR directed, weighted benchmark networks. Top: $\mu_t = \mu_w = 0.3$ (the number of planted communities is 35). Bottom: $\mu_t = \mu_w = 0.6$ (42 planted communities). See the text for the other parameters.

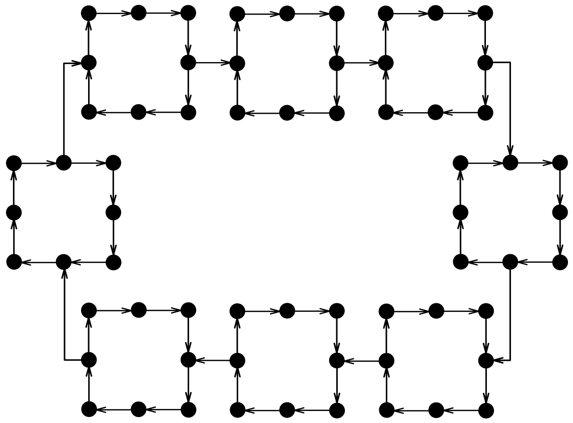

Figure S1.4. LinkRank benchmark network.

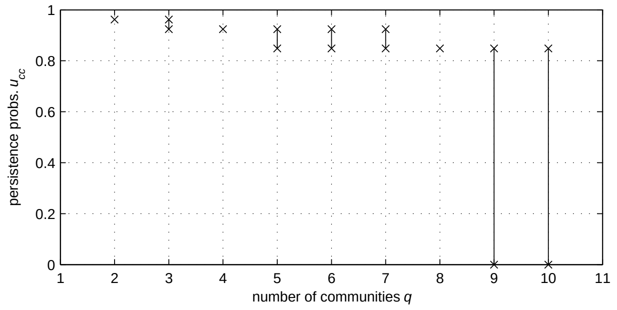

Figure S1.5. The persistence probabilities' diagram of the LinkRank benchmark network.

cyclic matrix U with all persistence probabilities equal to $u_{cc} = 0.8485$ (i.e., the probability of continuing to circulate in the same ring) and a unique non-zero off-diagonal entry $1 - u_{cc}$ in each row (i.e., the probability of jumping to the next ring).

Neural network

The last example concerns a directed, weighted network, representing the neural connections of the worm *Caenorhabditis elegans*. Starting from Watts and Strogatz's seminal work [9], different versions of this graph have become a standard benchmark for network analysis. We consider the directed, weighted version, whose largest connected component has $N = 239$ nodes.

Similarly to the case of the world trade network (see the main text), we show that our method is able to isolate well-defined communities even in a network which overall does not possess a definite clusterized structure. Consider the persistence probabilities' diagram of Fig. S1.6. With the exception of the trivial cases $q = 2$ and 3, no α-partition exists with α reasonably large. Nonetheless, a few α-communities with $\alpha \geq 0.8$ appear and are stably detected in a rather wide range of q. More precisely, the same set of five communities with $u_{cc} \geq 0.826$ are revealed in the range $14 \leq q \leq 20$. They are clusters, of dimension ranging from 18 to 29 nodes, with comparatively rather strong internal connectivity. Any other candidate cluster, instead, turns out to have a much smaller u_{cc} value and, therefore, it cannot be considered to be a meaningful community.

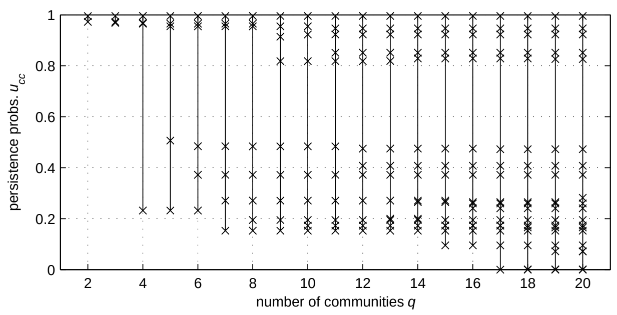

Figure S1.6. The persistence probabilities' diagram of the neural network.

Absolute and relative persistence probabilities

Several community detection methods (including max-modularity, as discussed in the main text) are based on a comparison between some quantity related to the network under scrutiny, and the same quantity computed on a randomized network (null model). Communities are thus significant if their structure markedly differ from the null model, which raises important questions on how to define a proper randomization (see, e.g., [2] for a discussion). Whereas the definition of u_{cc} does not contain such a sort of comparison, we can move in this line by complementing the "absolute" persistence probability u_{cc} with a *relative persistence probability* $r_{cc} = u_{cc} - Eu_{cc}$. Here Eu_{cc} is the value of the persistence probability of the same cluster $\mathbb{C}_c$ in a null model where the weight w_{ij} is replaced by its expected value, i.e., $Ew_{ij} = s_i s_j/(2S)$ for undirected networks, and $Ew_{ij} = s_i^{out} s_j^{in}/S$ for directed ones (we are adopting the same null model of modularity). By replacing these quantities in the expression of persistence probability (see main text), after some computation we obtain $Eu_{cc} = S_c/(2S)$ for undirected networks, and $Eu_{cc} = S_c^{in}/S$ for directed ones, where S_c^{in} is the sum of the in-strengths of the nodes of $\mathbb{C}_c$.

Plotting the r_{cc}-s gives a picture of the *relative* quality of each community/partition with respect to the null model. In Fig. S1.7, for example, the absolute and relative persistence probabilities are shown for the same network, namely a LFR benchmark having 38 built-in communities. Notice that, as it is typical, $\min_c u_{cc}$ is non-increasing with respect to q in all persistence probabilities' diagrams. On the contrary, the typical pattern we obtain when plotting the r_{cc}-s as functions of q shows an initial increase of $\min r_{cc}$, followed by a decrease. For small q, when clusters are very wide, even if the u_{cc}-s are large some of the Eu_{cc}-s are large too, yielding a small r_{cc}. On the other side, for large q some of the u_{cc}-s

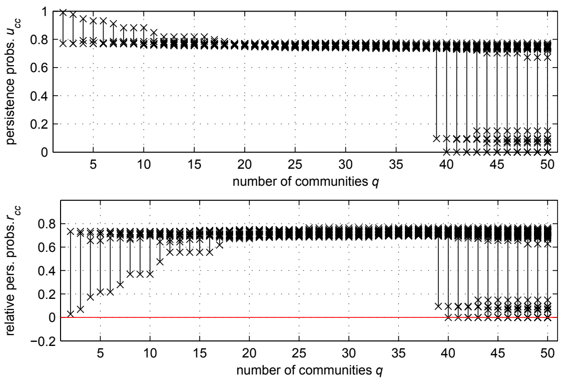

Figure S1.7. Absolute and relative persistence probabilities' diagrams of a LFR benchmark network. The relative persistence probability $r_{cc} = u_{cc} - Eu_{cc}$ compares the absolute one u_{cc} with the persistence probability Eu_{cc} of the same cluster in a null model.

rapidly drop, and so do the r_{cc}-s. A guideline, therefore, could be that of taking q such that $\min_c r_{cc}$ is maximized, as it yields the maximal differentiation of the clusters from the null model. This is an option, of course, although we point out that, for example, in the case portrayed in Fig. S1.7 such a maximum has scarce sensitivity, as it has almost the same value in $18 \leq q \leq 38$. Actually the maximum is attained for $33 \leq q \leq 37$, whereas the number of built-in communities is 38. But, apart from this, we think that the information conveyed by the absolute persistence probabilities u_{cc} is more representative: large clusters have naturally large u_{cc} values, whereas their r_{cc} is penalized just because their structure tends to be similar to that of the entire network (for which $u_{cc} = Eu_{cc} = 1$ and thus $r_{cc} = 0$). In other words, u_{cc} assigns a "quality value" to the cluster c, regardless to its size (even if so large to be scarcely meaningful), and leaves the network analyst the duty of selecting the desired trade-off between clusters' significance and size. In this respect, relative persistence probabilities do not seem to add value to the analysis, not counting that they are based on a specific choice of the null model (see [2] for a discussion on the criticalities in the definition of the null model).

References

1. Danon L, Diaz-Guilera A, Duch J, Arenas A (2005) Comparing community structure identification. J Stat Mech-Theory Exp : P09008.

2. Fortunato S (2010) Community detection in graphs. Phys Rep 486: 75-174.

3. Reichardt J, Bornholdt S (2006) When are networks truly modular? Physica D 224: 20-26.

4. Zachary W (1977) An information flow model for conflict and fission in small groups. J Anthropol Res 33: 452-473.

5. Weinan E, Li T, Vanden-Eijnden E (2008) Optimal partition and effective dynamics of complex networks. Proc Natl Acad Sci USA 105: 7907-7912.

6. Lancichinetti A, Fortunato S, Radicchi F (2008) Benchmark graphs for testing community detection algorithms. Phys Rev E 78.

7. Lancichinetti A, Fortunato S (2009) Benchmarks for testing community detection algorithms on directed and weighted graphs with overlapping communities. Phys Rev E 80.

8. Kim Y, Son SW, Jeong H (2010) Finding communities in directed networks. Phys Rev E 81: 016103.

9. Watts DJ, Strogatz SH (1998) Collective dynamics of 'small-world' networks. Nature 393: 440-442.
